# Myeloid-derived suppressor cells cross-talk with B10 cells by BAFF/BAFF-R pathway to promote immunosuppression in cervical cancer

**DOI:** 10.1007/s00262-022-03226-0

**Published:** 2022-06-20

**Authors:** Ding Jianyi, Gan Haili, Yin Bo, Yang Meiqin, Huang Baoyou, Hu Haoran, Li Fang, Zheng Qingliang, Han Lingfei

**Affiliations:** 1 Department of Gynecology, Shanghai First Maternity and Infant Hospital, Tongji University School of Medicine, Shanghai, 201240 China; 2 Department of Gynecology, School of Medicine, Shanghai East Hospital, Tongji University, Shanghai, 200120 China; 3 Sun Yat-sen University, Prenatal Diagnosis Center, The Eighth Affiliated Hospital, Shenzhen, 518000 People’s Republic of China

**Keywords:** Myeloid-derived suppressor cells (MDSCs), Interleukin (IL)-10-producing B cells (B10), BAFF, STAT3, Cervical cancer

## Abstract

Immunosuppression induced by myeloid-derived suppressor cells (MDSCs) is one of the main obstacles to the efficacy of immunotherapy for cervical cancer. Recent studies on the immunosuppressive ability of MDSCs have primarily focused on T cells, but the effect of MDSCs on B cells function is still unclear. In a study of clinical specimens, we found that the accumulation of MDSCs in patients with cervical cancer was accompanied by high expression of B cell activating factor (BAFF) on the surface and high expression of interleukin (IL)-10-producing B cells (B10) in vivo. We found that the absence of BAFF could significantly inhibit tumor growth in a cervical cancer model using BAFF KO mice. Further studies showed that abundant MDSCs in cervical cancer induced B cells to differentiate into B10 cells by regulating BAFF which acted on the BAFF receptor (BAFF-R) of them. In this process, we found that a large amount of IL-10 secreted by B10 cells can activate STAT3 signaling pathway in MDSCs, and then form a positive feedback loop to promote the differentiation of B10 cells. Therefore, this study reveals a new mechanism of BAFF-mediated mutual immune regulation between MDSCs and B cells in the occurrence and development of cervical cancer.

**Supplementary Information:**

The online version contains supplementary material available at 10.1007/s00262-022-03226-0.

## Introduction

In healthy individuals, immature myeloid cells (IMCs) migrate from the bone marrow to different peripheral organs where they differentiate into mature granulocytes, macrophages or dendritic cells. However, in cancer, various infectious diseases, sepsis, trauma, bone marrow transplantation, some autoimmune diseases and other pathological conditions, the differentiation of IMCs into mature myeloid cells is blocked, which leads to expansion of that cell population [1]. Under pathological conditions, these cells are activated and produce multiple immunosuppressive factors. In 2007, these cells were defined as bone marrow-derived suppressor cells [2]. In recent years, various data have shown that MDSCs are one of the main driving forces of the immunosuppressive tumor microenvironment (TME). The accumulation and activation of MDSCs are related to the progression, metastasis and recurrence of various types of tumors. Previous studies have shown that MDSCs can inhibit the acquired and natural antitumor immunity of the body through a variety of mechanisms [3, 4]. These include the production of arginase-1 (Arg-1), inducible nitric oxide synthase (INOS), indoleamine 2,3-dioxygenase (IDO) [5, 6] and a variety of soluble inhibitors that activate and amplify regulatory T cells (Tregs), so that tumor cells can escape immune surveillance, attack the body and promote tumor development. Some of these mechanisms have been confirmed in cancer patients [7–9].

Many studies on the immunosuppressive mechanism of MDSCs mainly focus on T cells and Treg cells, but the effects of MDSCs on B cells are still unclear. Studies have shown that B lymphocytes and T lymphocytes are mainly responsible for the basic functions of antibody production and cell-mediated immune response, respectively [10]. However, specific B cell subsets can also negatively regulate the immune response [11], and the presence of regulatory B cells (Bregs) has been verified [12–14]. Human and mouse Bregs that have the ability to produce the inhibitory cytokine IL-10 (B10 cells) have also been identified [14]. Although rare, B10 cells are effective negative regulators of antigen-specific inflammation and T cell-dependent autoimmune diseases in mice [15, 16]. However, the ways in which the production of IL-10 and the regulation of the antigen-specific immune response of B10 cells can be controlled in vivo are unknown. BAFF, as a member of the tumor necrosis factor family, is the key soluble factor in the maturation and survival of peripheral B cells [17]. BAFF is widely expressed in various cell types, including macrophages, dendritic cells and neutrophils [18]. Considering other researches on the role of MDSCs in B cell regulation [19–21], we speculate that BAFF may play a crucial role in the mechanism by which MDSCs regulate B cells to promote immune escape in cervical cancer.

In this study, we revealed the BAFF-mediated interaction between MDSCs and B cells. Through the interaction between BAFF/BAFF-R, MDSCs promote the proliferation of B cells and induce them to differentiate into B10 cells, which secrete a large amount of IL-10 that can activate the STAT3 pathway in MDSCs, thus forming a positive feedback pathway to further induce the differentiation of B10 cells, while the absence of BAFF on MDSCs can weaken these functions. These results show that BAFF plays an important role in regulating the interaction between MDSCs and B cells and participates in the progression of cervical cancer.

## Materials and methods

### Human blood samples

Whole blood samples collected at Shanghai First Maternity and Infant Hospital were obtained from patients with either malignant or benign tumors. All patients provided written consent. Peripheral blood mononuclear cells (PBMCs) were isolated by density gradient centrifugation (Ficoll Paque Plus; GE Healthcare, Pittsburgh, PA, USA) and used directly in experiments. The clinicopathological and demographic characteristics are listed in Table 1.

**Table 1:** Patients with malignant or benign tumors used to detect MDSCs in the PB

Group		Number		Age(years)
Malignant		37		56.15 ± 14.36
Benign		37		56.30 ± 11.80

### Animals

C57BL/6 mice were purchased from Shanghai SLAC Laboratory Animal Company and C57BL/6 BAFF KO mice were constructed by Suzhou Cyagen Company using a (CRISP)/CRISPR‐associated 9 (CAS9) system. Only female mice aged 6–8 weeks were used. All mice were maintained in a pathogen-free barrier facility at the Animal Experimental Center of Tongji University. The animal experiments were performed with the approval of the Institutional Laboratory Animal Care and Use Committee.

### Cell line

TC-1 tumor cells expressing the HPV-16 E6 and E7 proteins were obtained from American Type Culture Collection (ATCC) and were cultured in Roswell Park Memorial Institute (RPMI) 1640 medium supplemented with 10% fetal calf serum, as previously described.

### Flow cytometry

Human peripheral blood PBMC suspensions and single-cell suspensions prepared from mouse bone marrow, tumors and spleens were incubated with the following antibodies for 30 min at room temperature. For intracellular IFN-γ and IL-10 staining, a Cytofix/Cytoperm Fixation/Permeabilization Kit (554,714; BD Biosciences) was used. Anti-human:CD11b (FITC, eBioscience 11-0118-42), CD33 (APC, eBioscience 17-0338-42), HLADR (PerCP-Cyanine5.5, eBioscience 45-9956-42), CD14 (PerCP-Cyanine5.5, eBioscience 45-0149-42), CD15 (PE, Biolegend 301,906), BAFF (PE, eBioscience 12-9017-41), CD19 (PerCP-Cyanine5.5, eBioscience 45–0199-42), CD24 (FITC, eBioscience 11-0247-42), CD27 (PE, eBioscience 25-0279-42); anti-mouse: CD11b (APC, Biolegend 101,212), LY6G (FITC, eBioscience 11-9668-82), LY6C (PerCP-Cyanine5.5, eBioscience 45-5932-82), CD19(PerCP-Cyanine5.5, eBioscience 45-0193-82), CD1d (FITC, eBioscience), CD5 (APC, eBioscience 47-0051-82), IL-10 (FITC, eBioscience 11-7101-82), GR1 (PE, eBioscience 12-5931-82), CD3 (FITC, BD bioscience 555,274), CD4 (PE, BD bioscience 557,308), CD8 (APC, BD bioscience 553,035), IFN-*γ* (PerCP-Cyanine5.5, Biolegend 505,822). Finally, the stained cells were detected by flow cytometry (BD, FACSCalibur) and analyzed using FLOWJO 10.0 software (Becton, Dickinson & Company, Franklin Lakes, NJ, USA).

### Tumor model

TC1 cells in a logarithmic growth phase were washed with 1 × PBS 2–3 times and then counted under a microscope to observe whether the viability of tumor cells was suitable for inoculation. TC1 cells were transplanted subcutaneously into the right back of 6- to 8-week-old mice (1*10^6^/100 μl/mouse). Each group contained 3 mice. The survival time and tumor size were observed every 3 days. The calculation formula of tumor size is v = (a*b^2^), where a is the long diameter of tumor tissue and b is the short diameter of tumor tissue. After 28 days, the mice were sacrificed, and the spleen, tumor and femur tissues of tumor-bearing mice were gently collected for subsequent experiments.

### Cell separation

Mouse mononuclear cells were isolated by density gradient centrifugation (Ficoll Paque Plus; GE Healthcare, Pittsburgh, PA, USA). Mouse MDSCs were separated from spleen cells using a CD115 MicroBead Kit (Miltenyi Biotec, Cat. No. 130–096-354, Bergisch Gladbach, Germany) and MS column. The purity of the isolated cell population was determined using flow cytometry, and the frequency of CD11b^+^ GR1^+^ cells was > 85%.

### In vitro culture of murine cells

Mononuclear cells separated from naive mouse spleen were cultured alone or with MDSCs in the presence or absence of 10 μg/ml lipopolysaccharides (LPS) (Sigma, No. L4391-1MG) for 72 h; the ratio of nonadherent PBMCs to MDSCs was 3:1. Mouse mononuclear cells isolated from naive mouse spleens by density gradient centrifugation were cultured alone or with MDSCs in the presence or absence of 10 μg/ml mouse BAFFR/TNFRSF13C antibody or mouse IL-10 antibody (R&D Systems, Minneapolis, MN, USA, AF1357; MAB417). Mononuclear cells separated from naive mouse spleen were cultured alone or with MDSCs in the presence or absence of 5 μM WP1066 (MCE, No. HY-15312) or 0.5 μM colivelin (MCE, No. HY-P1061).

Finally, the differentiation ratio of B10 cells induced by MDSCs was detected by FACS, and the phenotype of B10 cells was CD19^+^ CD1d^+^ CD5^+^.

### Cytokine detection

The concentrations of INOS, IDO, ARG1 and IL-10 in human peripheral blood serum and the concentrations of INOS and IL-10 in the coculture supernatant of mouse B cells in vitro were detected with ELISA kits (R&D Systems, Minneapolis, MN, USA; Abcam, USA; Yuchunbio, China; Absin, China).

### Western blot

Total proteins were extracted using radioimmunoprecipitation assay extraction reagents (Cat. No. WB0102; WEIAO, Shanghai, China). A total of 8 μg of protein was separated by 10% sodium dodecyl sulfate polyacrylamide gel electrophoresis and subsequently transferred to polyvinylidene difluoride membranes. The membranes were blocked with 5% bovine serum albumin for 1 h at room temperature, incubated with the appropriate primary antibody at 4 °C overnight, and subsequently incubated with horseradish peroxidase-labeled secondary antibody for 1 h at room temperature before detection by enhanced chemiluminescence plus kit (Cat. WBULS0500; Millipore, Billerica, MA, USA). *β*-actin was used as an internal control for protein loading and analysis. The primary antibodies include anti-signal transducer and activator of transcription 3 (STAT3; Cat.4904 T; Applied at 1:1000; Cell Signaling Technology, USA), and anti-phosphorylated (p)-STAT3 (p-STAT3; Cat.9145 T; Applied at 1:1000; Cell Signaling Technology, USA). The secondary antibodies were horseradish peroxidase-conjugated anti-rabbit antibodies (Applied at 1:2000; Cell Signaling Technology, USA).

### Statistical analysis

All experiments were repeated at least three times in duplicate. Statistical analyses were performed by SPSS 20.0 (SPSS, Inc.) and GraphPad Prism 7.03 (GraphPad Software, Inc.). Data are shown as the mean ± SD. Differences between treated and control groups were analyzed using Student’s t test, nonparametric test and log-rank (Mantel–Cox) test. *P* values ≤ 0.05 were considered significant.

## Results

### Accumulation of MDSCs in cervical cancer patients

**Figure. 1:**
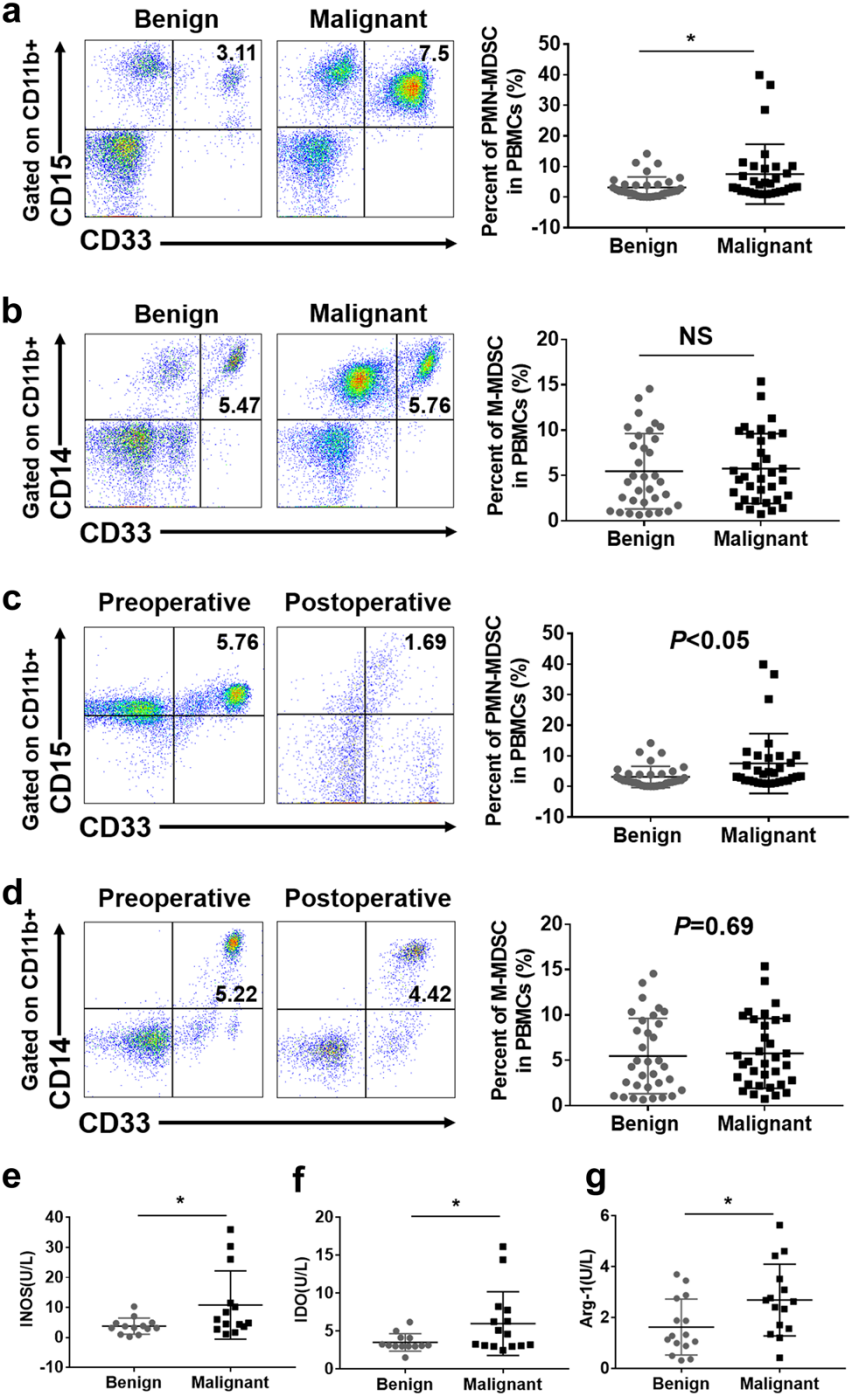
Accumulation of MDSCs in cervical cancer patients. **a** Flow cytometry analysis of PMN-MDSCs in peripheral blood from patients with benign or malignant tumors. Statistical analysis of the frequencies of PMN-MDSCs as a percentage of the mononuclear cells (MCs) population. **b** Flow cytometry analysis of M-MDSCs in peripheral blood from patients with benign or malignant tumors. Statistical analysis of the frequencies of M-MDSCs as a percentage of the MCs population. **c** Flow cytometry analysis of the frequency of PMN-MDSCs in the peripheral blood of patients with cervical cancer before and 5 days after surgery. Statistical analysis of the frequencies of PMN-MDSCs as a percentage of the MCs population. **d** Flow cytometry analysis of the frequency of M-MDSCs in the peripheral blood of patients with cervical cancer before and 5 days after surgery. Statistical analysis of the frequencies of M-MDSCs as a percentage of the MCs population. **e**–**g** ELISA analysis of the concentrations of IDO, INOS and Arg-1 in the peripheral blood of patients with either malignant or benign cervical tumors. Each point corresponds to an individual patient. Lines indicate the 25th to 75th percentiles. Horizontal lines represent the median value. Data were analyzed using Student’s t test and are expressed as the mean ± SD. Symbols represent statistical significance (*p < 0.05)

Our study first evaluated the accumulation of MDSCs in the peripheral blood of patients with cervical cancer and their immunosuppressive effect. And the information of clinical specimens involved was briefly described (Table. S1). The presence of PMN-MDSCs (polymorphonuclear myeloid-derived suppressor cells) and M-MDSCs (monocytic myeloid-derived suppressor cells) was detected in the peripheral blood of patients with benign or malignant tumors by flow cytometry. The phenotype of PMN-MDSCs was CD11b ^+^ CD33 ^+^ CD15^+^, while the phenotype of M-MDSCs was CD11b ^+^ CD33 ^+^ CD14^+^ [22]]. Results showed that the frequency of PMN-MDSCs in the peripheral blood of patients with cervical cancer was significantly higher than that in patients with benign tumors (3.105 ± 0.5986, *n* = 34 vs. 7.501 ± 1.729, *n* = 32, *p* < 0.05) (Fig. 1a). No significant difference was observed in the proportion of M-MDSCs (5.47 ± 0.7232, *n* = 33 vs. 5.757 ± 0.6604, *n* = 34, *p* > 0.05) (Fig. 1b).

In addition, we found that the frequency of PMN-MDSCs in the peripheral blood of patients with cervical cancer after operation was significantly lower than that before operation (5.758 ± 1.853, *n* = 6 vs. 1.686 ± 0.6584, *n* = 6, *p* < 0.05) (Fig. 1c); however, no significant difference was found in the frequency of M-MDSCs before and after operation (5.224 ± 1.465, *n* = 6 vs. 4.421 ± 1.452, *n* = 6, *p* > 0.05) (Fig. 1d).

Moreover, we detected the expression of INOS, IDO and Arg-1 by ELISA in the peripheral blood serum of patients with malignant or benign tumors, which are immunosuppressive factors secreted by MDSCs. The results showed that the concentrations of INOS, IDO, ARG-1 in the peripheral blood serum of patients with malignant tumors was significantly higher than that with benign tumors (Fig. 1e-g). These results suggest that a large number of PMN-MDSCs accumulate in the peripheral blood of cervical cancer patients, resulting in an immunosuppressive environment by secreting abundant immunosuppressive factors, which was relieved after operation.

### MDSCs with high BAFF expression and B10 cells secreting abundant IL-10 indicate the poor prognosis in cervical cancer

**Figure. 2:**
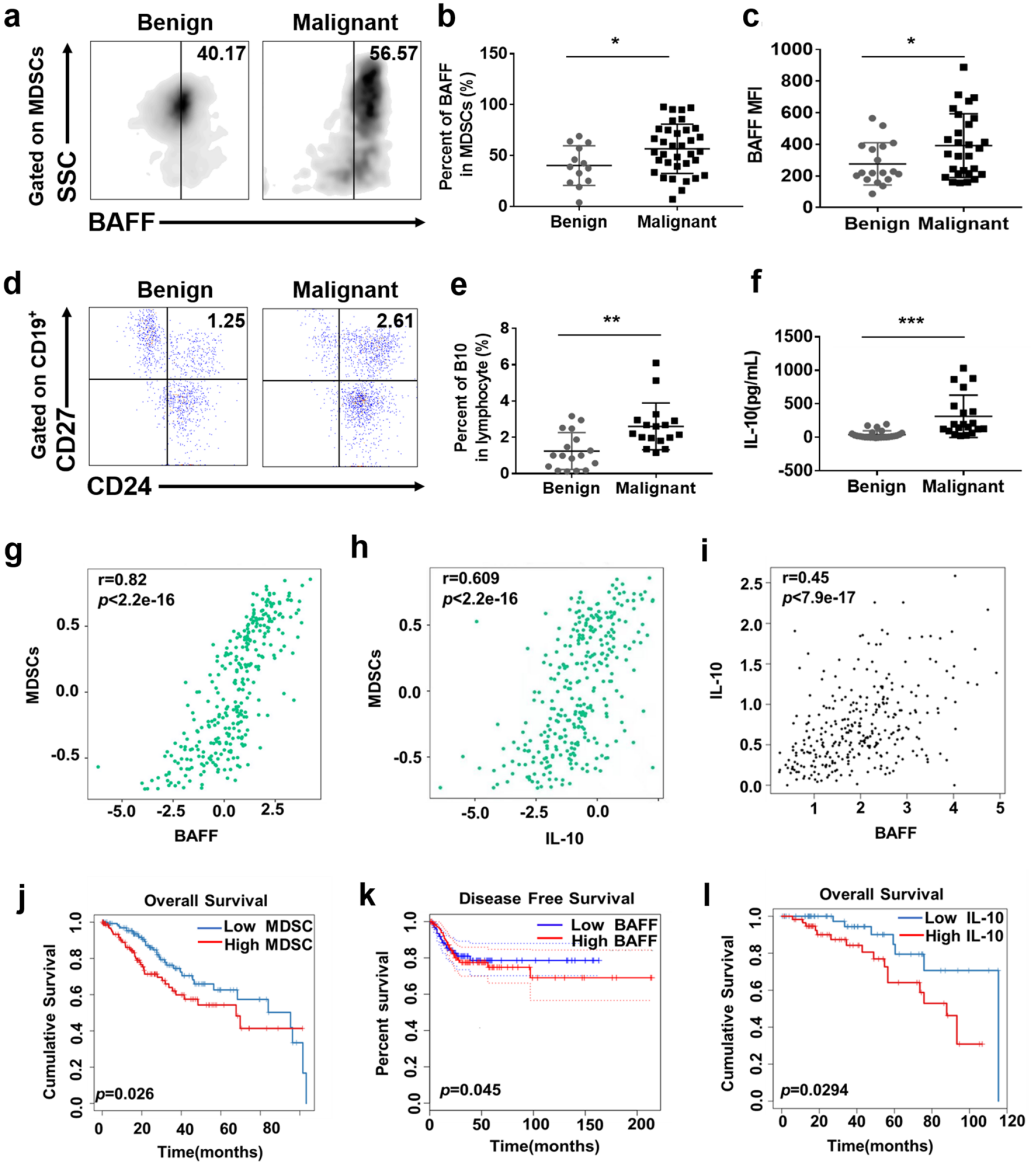
MDSCs with high BAFF expression and B10 cells secreting abundant IL-10 indicate the poor prognosis in cervical cancer. **a** Peripheral blood samples were collected from patients with malignant or benign cervical tumors, after which PBMCs were isolated by density gradient centrifugation (Ficoll Paque Plus). CD11b, CD33 and HLADR flow cytometry antibodies were used to gate MDSCs (CD11b^+^ CD33^+^ HLADR^−^) in PBMCs, and the BAFF-positive gate was set in MDSCs according to the isotype control antibody of BAFF. The positive expression of BAFF on the surface of MDSCs was gated. **b** Statistical analysis of the frequencies of BAFF expression in MDSCs in PBMCs. **c** CD11b, CD33 and HLADR flow cytometry antibodies were used to gate MDSCs (CD11b^+^ CD33^+^ HLADR^−^) in PBMCs, and the MFI value of BAFF on MDSCs was detected by flow cytometry. **d** Peripheral blood samples were collected from patients with malignant or benign cervical tumors, and PBMCs were isolated by density gradient centrifugation. CD19-positive cells were first gated in PBMCs, and then CD24 and CD27 were used to gate B10 cells (CD19^+^ CD24^+^ CD27^+^). **e** Statistical analysis of the frequencies of B10 cells as a percentage of the PBMCs population. **f** ELISA analysis of the IL-10 concentration in the peripheral blood of patients with malignant or benign cervical tumors. **g**–**i** Correlation analysis between MDSCs and BAFF and between MDSCs and IL-10 and between BAFF and IL-10 from GEPIA and TISIDB website. **j**, **l** The infiltration of MDSCs or IL-10 in 306 cervical cancer tissues was analyzed by the RNA-sq profile data in TIMER website, and further survival analysis of the association of MDSCs or IL-10 expression and overall survival in the cervical cancer was performed. **K** The expression of BAFF in 306 cervical cancer tissues were analyzed by the RNA-sq profile data in GEPIA website, and further survival analysis of the association of BAFF expression and disease-free survival in the cervical cancer was performed. Each point corresponds to an individual patient. Lines indicate the 25th to 75th percentiles. Horizontal lines represent the median value. Data were analyzed using Student’s t test and are expressed as the mean ± SD. Symbols represent statistical significance (*p < 0.05)

As mentioned above, BAFF, as the most critical factor in the maturation of B cells, is widely expressed in various cell types, including macrophages, dendritic cells and neutrophils. According to the origin of MDSCs, we speculated whether BAFF is expressed on the surface of MDSCs and associated with the occurrence and development of cervical cancer. To verify this hypothesis, we detected the expression of BAFF on MDSCs in the peripheral blood of patients with either benign (*n* = 13) or malignant cervical tumors (*n* = 35) by flow cytometry. The results showed that the expression of BAFF on MDSCs in the peripheral blood of patients with cervical cancer was higher than that with benign tumors (56.57 ± 4.088 vs. 40.17 ± 5.409, *p* < 0.01) (Fig. 2a, b). We also detected the mean fluorescence intensity (MFI) value of BAFF on MDSCs and found that the MFI value of the malignant group was significantly higher than that of the benign group (Fig. 2c).

Next, the proportion of B10 cells in peripheral blood samples of patients was evaluated and the results showed that the proportion of B10 cells in patients with malignant tumors was significantly higher than that with benign tumors (2.606 ± 0.3128, *n* = 17 vs. 1.245 ± 0.2463, *n* = 17, *p* < 0.01) (Fig. 2d, e). Furthermore, we found that the concentration of IL-10 in the peripheral blood serum of patients with cervical cancer was significantly higher than that with benign tumors (313.8 ± 70.76, *n* = 20 vs. 40.36 ± 11.51, *n* = 25, *p* < 0.001) (Fig. 2f).

In addition, the correlation between MDSC and BAFF or IL-10 was analyzed through TISIDB database, while the correlation between BAFF and IL-10 was analyzed through GEPIA database. The results confirmed that there is a positive correlation between them (Fig. 2g–i) [23, 24]. More importantly, we also found that increased MDSCs accumulation, IL-10 or BAFF expression independently correlates with shorter survival in cancer patients, which was supported by TIMER and GEPIA database (Fig. 2j–l) [24, 25]. Together, these data indicated that increased MDSCs accumulation, IL-10 or BAFF expression may participate in cervical cancer progression.

### Absence of BAFF inhibits tumor growth and decreases B10 cells and MDSCs

**Figure. 3:**
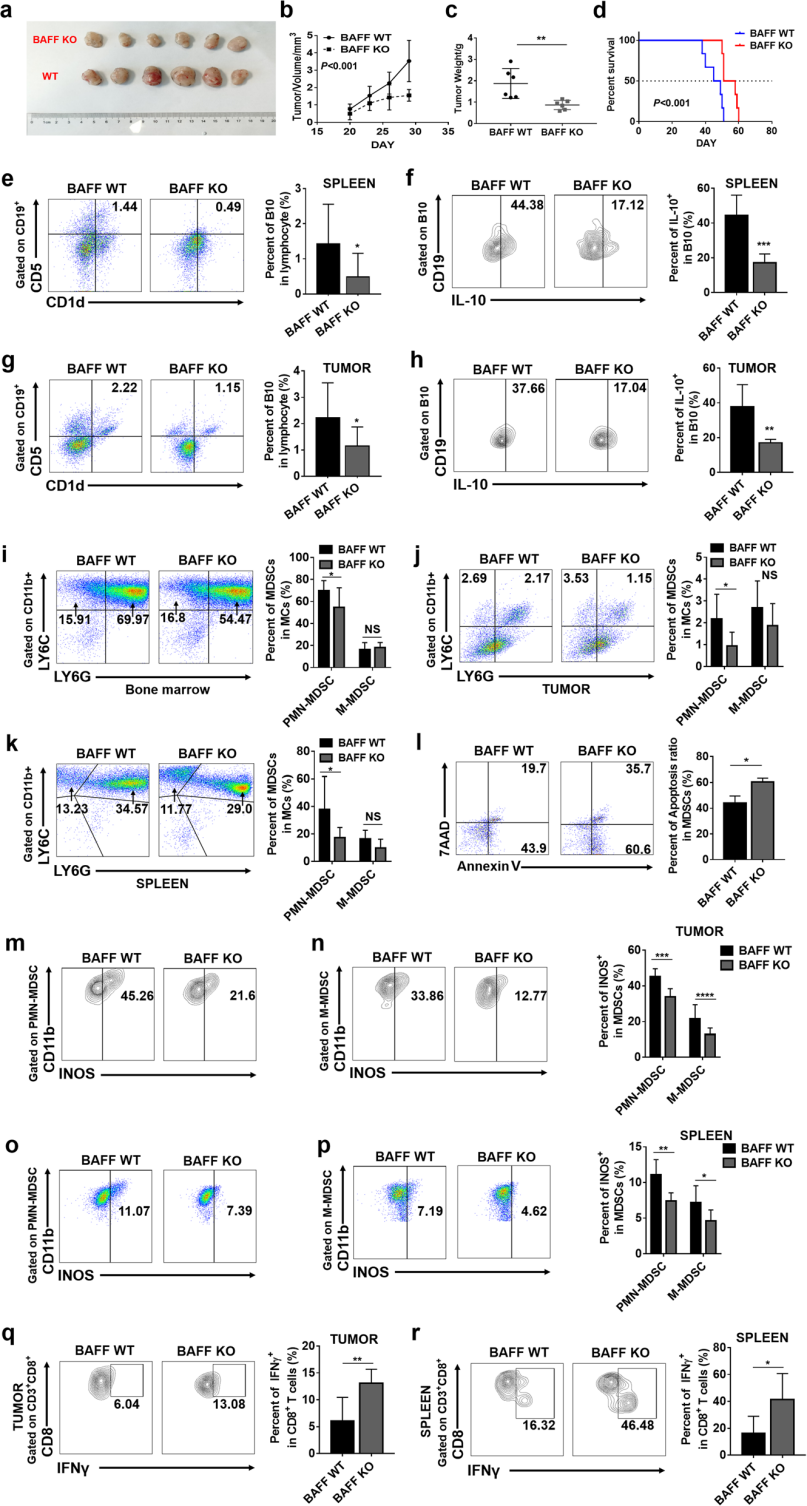
The absence of BAFF inhibited tumor growth and decreased the numbers of MDSCs and B10 cells. **a** TC1 cells were transplanted subcutaneously into the right back region of 6- to 8-week-old mice (1*10^6^/100 μl/mouse). The survival time and tumor size were observed every 3 days. **b**, **c** Mice were sacrificed on the 28th day of tumor growth, at which point the tumor was removed for measurement and weighing. **d** The probability of overall survival was significantly different in BAFF WT and BAFF KO groups. Data was analyzed using log-rank (Mantel–Cox) test. **e** Spleen single-cell suspension was prepared and mononuclear cells were isolated for flow cytometry. The CD19-positive cells were first gated in lymphocytes, and then, CD1d and CD5 were used to gate B10 cells (CD19^+^ CD1d^+^ CD5^+^). Statistical analysis of the frequencies of B10 cells as a percentage of the single-cell suspension. **f** B10 cells in the spleen were first gated and then the percentage of IL-10^+^ B10 cells were detected. **g** Tumor single-cell suspension was prepared and mononuclear cells were isolated for flow cytometry. The CD19-positive cells were first gated in lymphocytes, and then, CD1d and CD5 were used to gate B10 cells (CD19^+^ CD1d^+^ CD5^+^). Statistical analysis of the frequencies of B10 cells as a percentage of the single-cell suspension. **h** B10 cells in the tumor were first gated and then the percentage of IL-10^+^ B10 cells were detected. **i**–**k** Single-cell suspension of tumor, spleen and bone marrow were prepared. The CD11b-positive cells were first identified, and then, PMN-MDSCs and M-MDSCs were gated. Statistical analysis of the frequencies of PMN-MDSCs and M-MDSCs as a percentage of the MDSCs population. **l** The percentage of Annexin V^+^ MDSCs represents the proportion of apoptotic MDSCs. **m**–**p** PMN-MDSCs and M-MDSCs were first gated, and then, the percentage of INOS^+^MDSCs were detected. **q**–**r** Representative IFN-*γ*^+^ CD8^+^ T cells flowchart are shown in tumor and spleen tissues from cervical cancer models. Data from at least nine independent experiments were analyzed using Student’s t test and are expressed as the mean ± SD. Symbols represent statistical significance (*p < 0.05)

It has been demonstrated that BAFF can induce and promote B cells to differentiate into B10 cells with negative immunoregulation. We found that BAFF is highly expressed on MDSCs in cervical cancer patients, which correlates with high amounts of B10 cells. Therefore, we speculated whether MDSCs participated in the process of cervical cancer development by regulating BAFF to induce the differentiation of B cells into B10 cells. To verify this hypothesis, BAFF knockout C57BL/6 mice were used to established BAFF KO cervical cancer model by subcutaneous injection of the TC1 cell line, while wild-type (WT) C57BL/6 mice were injected with TC1 as the control. The tumor size was recorded and tumor weight was measured three weeks after TC1 injection. The results indicated that the tumor size and weight in BAFF KO mice group decreased compared with those in WT mice group (Fig. 3a, b, c). Furthermore, the probability of overall survival in BAFF KO group was significantly higher than that in WT group (Fig. 3d). Clearly, the loss of BAFF can significantly inhibit tumor growth.

Then, we detected the presence of B10 cells (CD19^+^ CD1d^+^ CD5^+^) in the spleen and tumors by flow cytometry [26]. The results showed that the presence of B10 cells in the spleen and tumors of BAFF KO tumor-bearing mice was significantly lower than that in WT mice accompanied with a weakened ability of secreting IL-10 (Fig. 3e–h). We also detected the frequency of MDSCs in bone marrow, spleen and tumor tissues by flow cytometry. The results showed that the proportion of PMN-MDSCs (CD11b^+^ LY6G^+^ LY6C^low^) in bone marrow, spleen and tumor tissue of BAFF KO tumor-bearing mice was significantly decreased compared with that in WT mice, while no significant difference was observed in M-MDSCs (CD11b^+^LY6G^−^ LY6C^high^) [22] (Fig. 3i–k).

Further studies found that compared with MDSCs derived from WT tumor bearing mice, the apoptosis of MDSCs in BAFF KO tumor bearing mice was significantly increased, which may explain why the proportion of MDSCs in BAFF KO tumor bearing mice decreased significantly (Fig. 3l). Next, in order to compare the immunosuppressive activity of MDSCs in the two groups of tumor-bearing mice, we tested the ability of spleen and tumor derived MDSCs of tumor bearing mice to secrete immunosuppressive factor INOS. The results showed that compared with WT tumor bearing mice, the ability of PMN-MDSCs and M-MDSCs from BAFF KO tumor bearing mice to secrete INOS decreased significantly (Fig. 3m-p).

Moreover, by detecting the proportion of CD8^+^ IFN*γ*^+^ T cells in the spleen and tumor of the two groups of tumor-bearing mice, we found that the ability of CD8^+^ T cells derived from BAFF KO tumor bearing mice to secrete IFN*γ* was significantly increased compared with WT tumor bearing mice (Fig. 3q, r). Taken together, BAFF deletion can reduce B10 cells and PMN-MDSCs in tumor bearing mice. Although the proportion of M-MDSCs has not changed in two groups, the absence of BAFF weakened its immunosuppressive activity, indicating that the absence of BAFF affects the function of both types of MDSCs.

### MDSCs induces B10 cell differentiation through BAFF/BAFF-R pathway

**Figure. 4:**
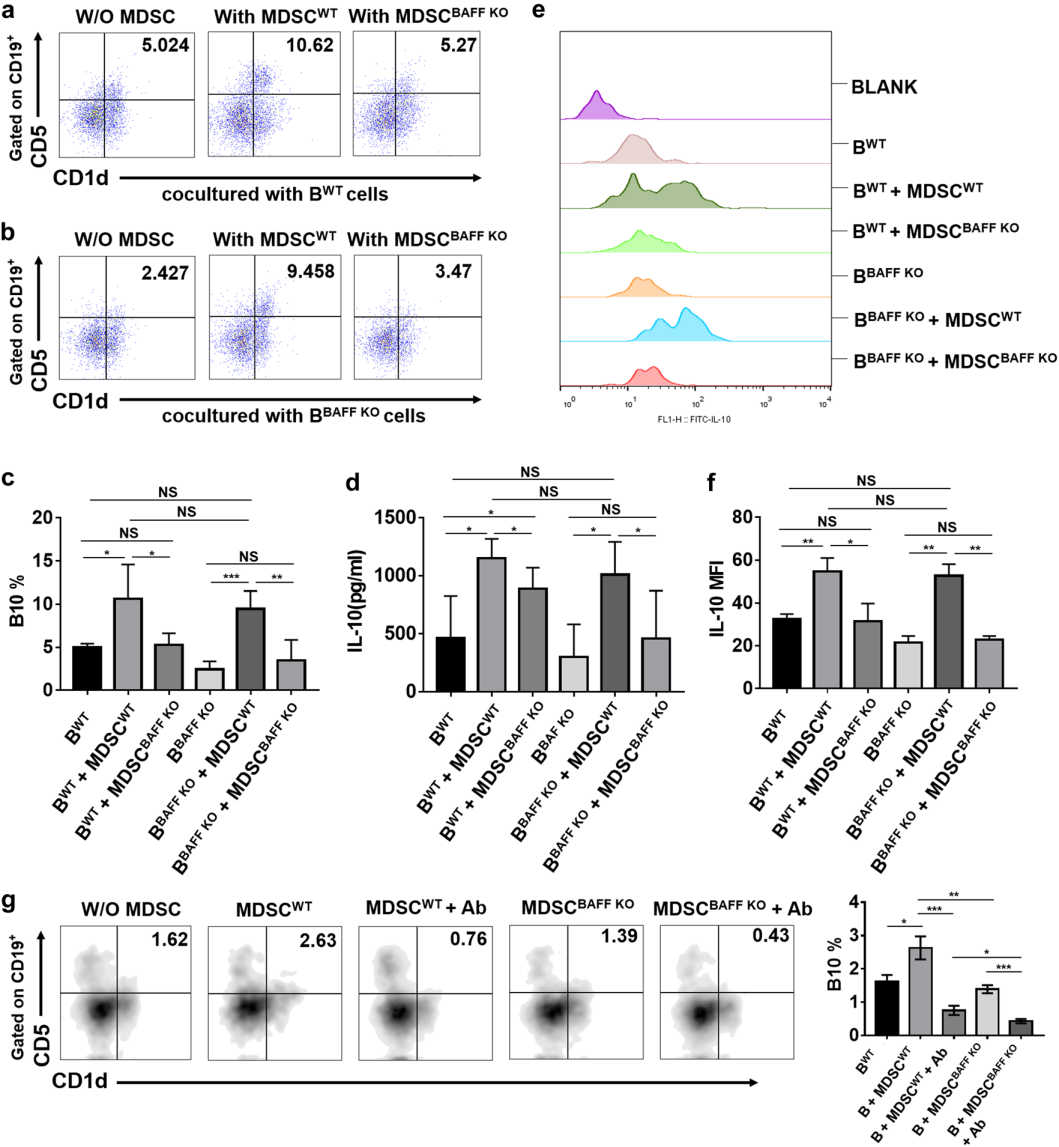
MDSCs induce B10 cell differentiation through BAFF/BAFF-R pathway. **a**, **b** Mouse MCs isolated by density gradient centrifugation were cocultured with MDSCs at a ratio of 1:3 for 72 h with LPS (1 µg/ml) stimulation. The differentiation ratio of B10 cells was detected by flow cytometry. **c** Statistical analysis of B10 differentiation in vitro. **d** The concentration of IL-10 in the coculture supernatant was detected by ELISA. **e** CD19, CD1d and CD5 flow cytometry antibodies were used to gate B10 cells (CD19^+^ CD1d^+^CD5^+^) in PBMCs, and the MFI value of IL-10 on B10 cells was detected by flow cytometry. **f** Statistical analysis of the MFI value of IL-10 on B10 cells. **g** Mouse MCs isolated by density gradient centrifugation were cocultured with MDSCs in the presence or absence of anti-BAFF-R Ab. The differentiation ratio of B10 cells was detected by flow cytometry. Data from at least three independent experiments were analyzed using Student’s t test and are expressed as the mean ± SD. Symbols represent statistical significance. (*p < 0.05; **p < 0.001; ***p < 0.0001)

According to the in vivo experimental results, we found that BAFF deficiency can affect the accumulation and function of both MDSCs and B10 cells. To further verify the effect of BAFF on MDSCs and B10 cells, we conducted in vitro experiments. These two groups of spleen-derived MDSCs were cocultured with mononuclear cells isolated from the spleens of BAFF KO and WT naive mice under the LPS (1 µg/ml) stimulation. The differentiation ratio of B10 cells was detected. We found that when cocultured with B^WT^ cells, MDSC^WT^ could significantly promote the differentiation of B10 cells compared with MDSC^BAFF KO^ or B^WT^ alone, but no difference was detected between MDSC^BAFF KO^ cocultured with B^WT^ and B^WT^ alone, and the same results were also obtained in the coculture with B^BAFF KO^ (Fig. 4a, b, c).

Furthermore, we also found no significant difference in the proportion of B10 cell differentiation between MDSC^WT^ cocultured with B^WT^ and MDSC^WT^ cocultured with B^BAFF KO^ or between MDSC^BAFF KO^ cocultured with B^WT^ and MDSC^BAFF KO^ cocultured with B^BAFF KO^ (Fig. 4c). What is more, our results suggest that B cells in each group can hardly differentiate into B10 without the stimulation of LPS (Figure. S1a, b, c).

According to previous studies, B10 cells can secrete IL-10[17]. Thus, we detected the concentration of IL-10 in the coculture supernatant by ELISA; the results were consistent with the results of coculture-induced B10 cell differentiation. (Fig. 4d). For further verification, we detected the MFI of IL-10 on B10 cells in the coculture system by flow cytometry, and obtained the same results as in ELISA (Fig. 4e, f).

To further confirm that BAFF induces B cell differentiation by acting on the BAFF receptor (BAFF-R) of B cells, we performed a group of neutralizing antibody experiments against BAFF-R and found that MDSCs did not induce B cell differentiation into B10 cells in the presence of a neutralizing antibody against BAFF-R (BAFF-R Ab) (Fig. 4g). The above results show that MDSCs could induce B cells to differentiate into B10 cells and promote B10 cells to secrete IL-10 by BAFF on the surface of MDSCs, which functions through BAFF receptors on B cells.

### IL-10 derived from B10 activates MDSCs through the STAT3 pathway

In vivo, we found that the tumor size and weight of BAFF knockout mice decreased compared with those of WT mice and the lower frequency of B10 cells was also accompanied with a decrease of MDSCs. Therefore, we speculate whether B10 cells will also affect the function of MDSCs. Based on this conjecture, we designed a vitro experiment: MDSCs isolated from WT or BAFF KO tumor bearing mice were cultured in three groups: MDSCs cultured alone, MDSCs co-cultured with B cells from WT mice, and MDSCs co-cultured with B cells from BAFF KO mice. After 72 h of culture in the presence of LPS, the supernatants of each group were collected and concentration of INOS was detected by ELISA.

**Figure. 5:**
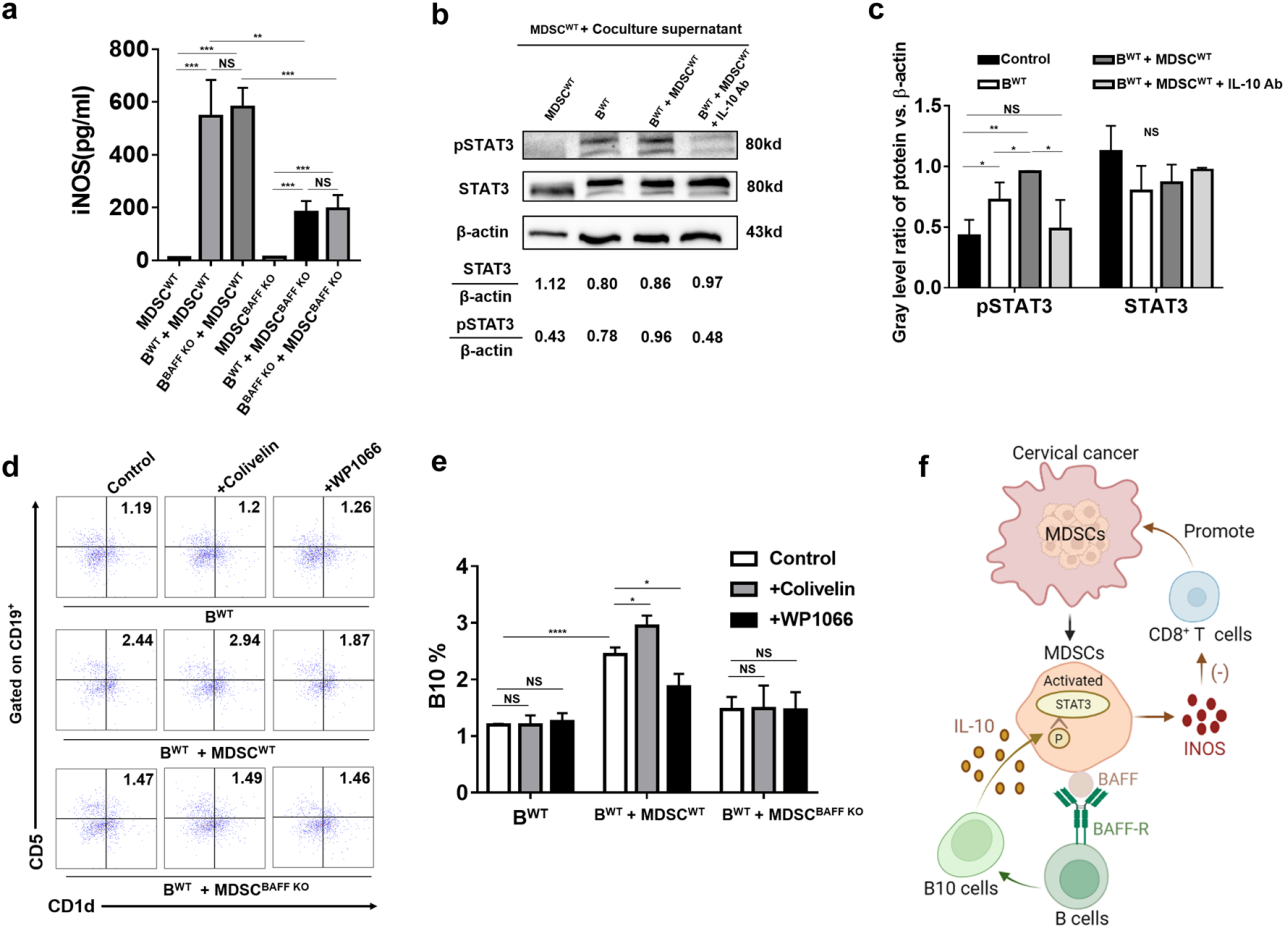
IL-10 derived from B10 activates MDSCs through the STAT3 pathway. **a** MDSCs were isolated from the spleens of the two groups of tumor-bearing mice on day 28 by CD115 magnetic beads and cocultured with mouse MCs isolated by density gradient centrifugation from BAFF WT and BAFF KO mouse spleens at a ratio of 1:3 for 72 h or alone, under the stimulation of LPS (1 µg/ml). The concentration of INOS in the coculture supernatant was detected by ELISA. **b** The culture supernatant of B^WT^, MDSCs cocultured with B^WT^, MDSCs cocultured with B.^WT^ in the presence of IL-10 neutralizing antibody was collected and then cocultured with MDSCs for 48 h. The STAT3 phosphorylation level of MDSCs was detected by western bolt. **c** Statistical analysis of the STAT3 phosphorylation level of MDSCs. **d** Mouse MCs isolated by density gradient centrifugation were cocultured with MDSCs at a ratio of 1:3 for 72 h with or without WP1066 or colivelin stimulation. The differentiation ratio of B10 cells was detected by flow cytometry. **e** Statistical analysis of B10 differentiation in vitro. **f** Graphical abstract of MDSCs cross-talk with B10 cells by BAFF/BAFF-R pathway to promote immunosuppression in cervical cancer. Data from at least three independent experiments were analyzed using Student’s t test and are expressed as the mean ± SD. Symbols represent statistical significance. (*p < 0.05; **p < 0.01; ***p < 0.001; ****p < 0.0001)

Interestingly, we found that compared with MDSCs alone, MDSCs produced more INOS in the coculture group (Fig. 5a). Moreover, in the coculture system, INOS secretion in the supernatant of the MDSC^BAFF KO^ groups was significantly reduced (Fig. 5a). This evidence indicates that B cells can induce MDSCs to secrete a high level of inhibitory factor INOS and the absence of BAFF on MDSCs can weaken their ability to secrete inhibitory factor INOS. These results indicated that there must be a two-way regulation system between MDSCs and B cells depending on BAFF.

Many studies have proved that IL-10 derived from cancer can activate MDSCs through STAT3 pathway [4], and in our study, we found that MDSCs are accompanied by a large amount of IL-10 secretion in the process of inducing B10 cell differentiation. Therefore, we collected the culture supernatant of B^WT^, MDSCs cocultured with B^WT^, MDSCs cocultured with B^WT^ in the presence of IL-10 neutralizing antibody, and then cocultured with MDSCs for 48 h to detect the STAT3 phosphorylation level of MDSCs. And we found that compared with MDSCs blank, supernatant from B^WT^ or MDSCs cocultured with B^WT^ could significantly promote STAT3 phosphorylation in MDSCs, and the level of STAT3 phosphorylation induced by MDSCs cocultured with B^WT^ supernatant was more significant, which could be weakened by IL-10 neutralizing antibody (Fig. 5b, c).

Then, we used STAT3 pathway activators and inhibitors to verify whether the activation of STAT3 pathway in MDSCs is involved in its regulation of B10 cell differentiation. The results showed that after using STAT3 pathway inhibitor-wp1066 or STAT3 pathway activator-colivelin in the MDSC^WT^ cocultured with B^WT^ group, it could significantly reduce or enhance MDSC^WT^ ability to induce B10 cell differentiation, but the same phenomenon was not detected in B^WT^ alone or MDSC^BAFF KO^ cocultured with B^WT^ group (Fig. 5d, e). Based on the above results, we concluded that IL-10 secreted by B10 cells activates STAT3 pathway in MDSCs, which further regulates the differentiation of B10 cells depending on the BAFF on the surface of MDSCs.

Taken together, these results suggest that MDSCs induce B cells to differentiate into B10 cells through BAFF/BAFF-R axis. Furthermore, IL-10 secreted by B10 cells can promote STAT3 phosphorylation in MDSCs and activate MDSCs. The activated MDSCs can secrete immunosuppressive factors, such as INOS, which then can inhibit CD8^+^ T cells, and finally realizes the immune escape of cervical cancer (Fig. 5f).

## Discussion

Cervical cancer is one of the most serious malignant tumors that threatens women’s health. Its incidence rate is second only to ovarian cancer and cancer of the uterine body. In recent years, its incidence rate and mortality rate have increased. We found that the immune system of patients with cervical cancer is in a state of immune incompetence, which leads to the failure of HPV vaccines and immunotherapy. In our research, we found that MDSCs accumulated in cervical cancer patients and secreted abundant immunosuppressive factors, which indicates that MDSCs are one of the main driving forces of immunosuppression in the tumor microenvironment [27–32]. MDSCs derived from various pathological conditions (such as cancer, inflammation and autoimmune diseases) can inhibit specific antitumor immunity mediated by T cells and natural antitumor immunity mediated by NK cells and macrophages by expressing high levels of ARG-1, INOS and ROS [5, 6]. The ability of MDSCs to inhibit T cells and induce the differentiation of Treg cells has been verified, but the exact role of MDSCs in B cells is not clear [33].

B cells, which are defined by their humoral function of secreting antibodies, play an important role in the activation of CD4^+^ T cells and the development of lymphoid tissue structures. However, historically, B cells have been considered to be key negative regulators of normal and abnormal immune responses [34]. In recent years, investigators have attributed additional functions to B cells, including antigen presentation, the production of multiple cytokines and a suppressive capacity that is ascribed chiefly to their secretion of IL-10 [35, 36]. Convincing data have demonstrated that IL-10-producing B cells, termed regulatory B cells (Bregs) in 2002 by Bhan and collaborators [37], can suppress inflammatory responses in experimental autoimmune encephalomyelitis (EAE), collagen-induced arthritis (CIA) and colitis [38, 39]. Moreover, many studies have suggested that B10 cells that produce IL-10 can inhibit the antitumor effect of T cells and promote tumor growth [40, 41]. All this evidence indicates that B10 cells have a negative immunoregulatory effect and are involved in tumor growth. Consistent with these findings, our study found that the proportion of B10 cells in patients with malignant cervical cancer was significantly increased and that a high level of IL-10 was secreted.

BAFF is a member of the TNF family and is a key regulator of B cell maturation and survival. Evidence from BAFF transgenic mice shows that BAFF induces CD4^+^ Foxp3^+^ T cells to inhibit the T cell response in an indirect B cell-dependent manner, which suggests that BAFF has a regulatory role in vivo [41]. It has been demonstrated that BAFF can induce B10 cell differentiation and negative immune regulation [42]. It has also been confirmed that BAFF is expressed in various cell types, including macrophages, dendritic cells and neutrophils, which derived from IMCs, while MDSCs are the pathological state of IMCs. Our experiment verified that BAFF is highly expressed on MDSCs of patients with malignant cervical cancer. On this basis, we found that the absence of BAFF can reduce the aggregation of MDSCs and B10 cells and inhibit tumor growth. In addition, the absence of BAFF will lead to the weakening of the immunosuppressive function of MDSCs in tumor bearing mice, which is manifested in the weakening of the ability to secrete immunosuppressive factor INOS, accompanied by the increase of CD8^+^ IFN*γ*^+^ T cells. Interestingly, although the proportion of M-MDSCs does not seem to decrease in BAFF KO tumor bearing mice, its ability to secrete INOS is weakened, which is consistent with PMN-MDSCs, suggesting that BAFF deletion may lead to changes in the function of both types of MDSCs. Therefore, in next experiments, we did not explore the functions of PMN MDSCs and M-MDSCs separately. In functional experiments, we found that MDSCs promote the differentiation of B cells into B10 cells by regulating the binding of BAFF to BAFF-R on B cells. Interestingly, we also found that B cells could induce MDSCs to secrete high concentration of INOS in the coculture system; however, in the absence of BAFF expression on MDSCs, the phenomenon was attenuated. Further studies showed that IL-10 secreted by B10 cells could activate STAT3 pathway in MDSCs and further induce B10 cells differentiation in the presence of BAFF, which forms a positive feedback loop. This has not been mentioned in other studies. Obviously, BAFF plays a significant role in this mechanism, but we just detected that BAFF plays a role through BAFF-R receptor. Whether it affects immune cells through other ways needs further experimental verification. However, the current results suggest that BAFF may be an important biomarker in cervical cancer.

In general, we revealed that MDSCs and B cells could achieve a positive feedback loop by BAFF/BAFF-R/IL-10, which leads to the differentiation of B10 cells and the activation of MDSCs. This in turn induces an immunosuppressive state in patients with cervical cancer and provides an immune escape mechanism. This is of great scientific significance for the development of therapeutic vaccines and for improving the response rate of cervical cancer to immunotherapy.

## Supplementary Information

Below is the link to the electronic supplementary material.

Supplementary file1 (DOCX 13 kb)

Supplementary file2 (DOCX 81 kb)

## Abbreviations

Arg-1: Arginase-1
B10: Interleukin (IL)-10-producing B cells
BAFF: B cell activating factor
BAFF-R: BAFF receptor
Bregs: Regulatory B cells
IDO: Indoleamine 2,3-dioxygenase
INOS: Inducible nitric oxide synthase
IMCs: Immature myeloid cells
M-MDSCs: Monocytic myeloid-derived suppressor cells
MCs: Mononuclear cells
MDSCs: Myeloid-derived suppressor cells
PBMCs: Peripheral blood mononuclear cells
PMN-MDSCs: Polymorphonuclear myeloid-derived suppressor cells
TME: Tumor microenvironment
Tregs: Regulatory T cells
WT: Wild-type
