# Supplementary material for: Myeloid-derived suppressor cells cross-talk with B10 cells by BAFF/BAFF-R pathway to promote immunosuppression in cervical cancer
Source: Cancer Immunol Immunother. 2022 Jun 20;72(1):73–85. doi: 10.1007/s00262-022-03226-0 (PMC9813028; doi:10.1007/s00262-022-03226-0)
Supplement: Supplementary file 1 — Supplementary file1 (DOCX 13 kb) [file 262_2022_3226_MOESM1_ESM.docx]

| **Table. S1.** Patients with Malignant or Benign Tumors used to detect MDSCs in the PB. | | | |
| --- | --- | --- | --- |
| Group | Number | Age（Years） |  |
| Malignant | 37 | 56.15±14.36 |  |
| Benign | 37 | 56.30±11.80 |  |
