# Supplementary material for: Myeloid-derived suppressor cells cross-talk with B10 cells by BAFF/BAFF-R pathway to promote immunosuppression in cervical cancer
Source: Cancer Immunol Immunother. 2022 Jun 20;72(1):73–85. doi: 10.1007/s00262-022-03226-0 (PMC9813028; doi:10.1007/s00262-022-03226-0)
Supplement: Supplementary file 2 — Supplementary file2 (DOCX 81 kb) [file 262_2022_3226_MOESM2_ESM.docx]

Figure. S1


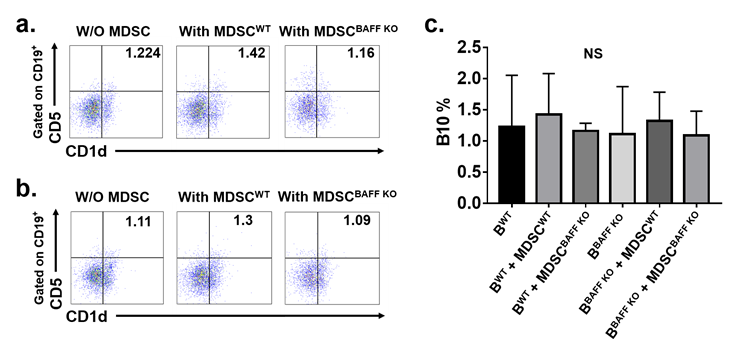


Figure. S1 The detection of the differentiation ratio of B10 cells without LPS stimulation (a) BAFF WT mouse MCs isolated by density gradient centrifugation were cocultured with MDSCs at a ratio of 1:3 for 72 h without LPS (1 µg/ml) stimulation. The differentiation ratio of B10 cells was detected by flow cytometry. (b) BAFF KO mouse MCs isolated by density gradient centrifugation were cocultured with MDSCs at a ratio of 1:3 for 72 h without LPS (1 µg/ml) stimulation. The differentiation ratio of B10 cells was detected by flow cytometry. (c) Statistical analysis of B10 differentiation in vitro.
